# Trajectory Planning of Flexible Walking for Biped Robots Using Linear Inverted Pendulum Model and Linear Pendulum Model

**DOI:** 10.3390/s21041082

**Published:** 2021-02-04

**Authors:** Long Li, Zhongqu Xie, Xiang Luo, Juanjuan Li

**Affiliations:** 1 School of Mechanical Engineering, Southeast University, Nanjing 211189, China; lilong@seu.edu.cn (L.L.); xiezq@seu.edu.cn (Z.X.); 2 School of Mechanical-Electrical Engineering, North China Institute of Science and Technology, Beijing 065201, China; lantianljj@163.com

**Keywords:** linear inverted pendulum model, linear pendulum model, single support phase, double support phase, biped robots, trajectory planning

## Abstract

Linear inverted pendulum model (LIPM) is an effective and widely used simplified model for biped robots. However, LIPM includes only the single support phase (SSP) and ignores the double support phase (DSP). In this situation, the acceleration of the center of mass (CoM) is discontinuous at the moment of leg exchange, leading to a negative impact on walking stability. If the DSP is added to the walking cycle, the acceleration of the CoM will be smoother and the walking stability of the biped will be improved. In this paper, a linear pendulum model (LPM) for the DSP is proposed, which is similar to LIPM for the SSP. LPM has similar characteristics to LIPM. The dynamic equation of LPM is also linear, and its analytical solution can be obtained. This study also proposes different trajectory-planning methods for different situations, such as periodic walking, adjusting walking speed, disturbed state recovery, and walking terrain-blind. These methods have less computation and can plan trajectory in real time. Simulation results verify the effectiveness of proposed methods and that the biped robot can walk stably and flexibly when combining LIPM and LPM.

## 1. Introduction

Compared with other types of robots, humanoid robots have good adaptability to the environment, stronger obstacle avoidance ability, and a smaller moving blind area, which has attracted the attention and in-depth research of scholars [1,2,3,4,5,6,7,8,9]. At present, biped robots are still quite far away from the real sense of anthropomorphism, and there are many problems to be solved in this field. For example, due to the inherent instability of biped walking, walking stability analysis is still an important issue for biped robots. In addition, the biped robot is a high-order and strong coupling nonlinear system, which makes the trajectory planning and control difficult. The realization of stable walking is the primary task in the research of humanoid robots.

There are many methods for gait planning of biped robots. These methods could be divided into two classes. The first uses the accurate information of dynamical parameters to generate walking patterns. Joint angle trajectories [10,11,12,13,14] or trajectories of some key parts [15,16,17,18], e.g., hip and/or feet, are usually fitted by spline or polynomial functions, then the coefficients of spline or polynomial functions are determined by parameter optimization technique. However, these gait-planning methods need a lot of computation and cannot meet the requirement of trajectory planning in real time. The more degrees of freedom and the higher the order of the polynomials, the more computation time is needed for solving the optimization problem.

The other class is based on a simplified model to generate walking patterns. Inverted pendulum [19,20] is widely used because of its simplicity. A biped robot is usually regarded as a concentrated mass and massless leg. The trajectory of the center of mass (CoM) is planned with a simplified model, and then the angles of other joints are solved by inverse kinematics. One of the widely used methods is the linear inverted pendulum model (LIPM) [21,22]. The advantage of LIPM is that the trajectory of the CoM has an analytical solution. Moreover, its forward and lateral motions are decoupled. Another model is the inverted pendulum model (IPM) with constant leg length [23,24,25]. In this model, the CoM moves along an arc. Although the dynamic equation of IPM is simple, there is no analytical solution due to its nonlinearity. The disadvantages of LIPM and IPM are that they can only generate the trajectory of the single support phase (SSP), but cannot generate the trajectory of the double support phase (DSP).

From an application perspective, when the biped robot is walking outdoors, due to the unstructured ground environment, the robot is required to have the ability of real-time gait generation according to the current environment. However, the more accurate the model is, the more computation is needed. Hence real-time gait planning may become very difficult. Therefore, the simplified model is a feasible and very useful method for real-time gait planning.

On the other end of spectrum, there is little attention on the DSP. Many gait-planning methods consider only the SSP and ignore the DSP, or the DSP is assumed to be instantaneous. In this situation, the center of pressure (CoP) or zero-moment point (ZMP) [2] needs to transfer from the trailing foot to the leading foot instantaneously when the support leg is switched. This requires an impulsive force between the rear foot and the ground. The emerge of impulsive force could lead to some adverse factors: (1) it has a negative effect on the walking stability analysis; (2) generating impulse force needs a sufficiently large joint torque that the joint driving motors may not provide; (3) it may damage the hardware of the robot. The introduction of the DSP can reduce the impact between the foot and the ground, make a smooth ZMP transition from the trailing foot to the leading foot, and improve walking stability. In addition, the support polygon area of the DSP is larger than that of the SSP, so the ground can provide greater external torque to the robot during the DSP. Therefore, the robot has stronger state-adjustment ability during the DSP. During the SSP, because the robot’s foot is small, the ground cannot provide a large enough external torque to avoid the robot falling down; as a result, the robot needs more adjustment of the internal state. Kajita and Tani reported that adding the DSP to the LIPM reduced the loss of the CoM’s velocity when the support leg exchanges [26].

To overcome shortcomings of models without the DSP, some scholars introduced the DSP in gait planning. Kajita et al. [27] planned the CoM’s trajectory of the DSP as a fourth-order polynomial function. The coefficients of the polynomial are determined by the boundary condition and the specified duration of the DSP. Motoi et al. [28] designed the CoM’s trajectory in the DSP as a fifth-order polynomial function. The disadvantage of their method is that walking stability was not taken into consideration during the DSP. With the increase of the order of the polynomial, unexpected oscillation of CoM may occur; as a result, unexpected oscillation of the ZMP may occur during the DSP. In addition, the displacement of the CoM during the DSP is not intuitive and cannot be perfectly integrated with LIPM.

Shibuya et al. [29] proposed the linear pendulum model (LPM) to plan the trajectory of the CoM in the DSP, and determine the appropriate suspension point, which can ensure that the acceleration of the CoM is continuous at the moment of the switch between the SSP and the DSP. However, they only plan the cyclic gait of the robot on the horizontal ground, and do not give the gait-planning method when the robot faces a more complex environment. Shibuya et al. [30] extended the results of [29] to generate DSP trajectories in two situations. One is to land the swing leg earlier than planned, and the other is trajectory planning to stop walking in the DSP. However, they still did not put forward the method in more situations.

In our previous work, the two-point-foot walking model was proposed. A planar walking pattern was designed in [31]. Then this method is extended to 3D biped walking in [32]. Furthermore, it was implemented to the speed control for dynamic walking [33]. None of these works have fully considered the role of the DSP. In this study, we extend our works and add a DSP in gait planning.

In order to meet the requirement of real-time trajectory generation in complex environments, the gait-planning method should satisfy either planning simplicity or walking stability. In this paper, LIPM and LPM are used to plan the trajectories of the SSP and the DSP, respectively. The dynamic equations of LIPM and LPM are linear, so they have analytic solutions. Trajectory planning only needs a small amount of computation. Through dynamic analysis of two pendulum models and their ZMP, the stability of gait can be guaranteed. Moreover, LPM is well-compatible with LIPM. Not only does the trajectory of CoM have an analytical solution, but the displacement of CoM in the SSP and DSP also has a very intuitive geometric representation.

The novelty of the paper is to propose a trajectory-planning method using LIPM and LPM, which can achieve flexible walking for biped robots. According to our proposed method, biped robots can generate trajectory online for several cases, such as periodic walking, changing walking speed, disturbance recovery, and walking on uneven ground, etc. The main difference between this paper and other papers is the trajectory-planning method in the double support phase. In some papers, the trajectory of the CoM is planned by LIPM in the SSP and is designed as a polynomial in the DSP. However, the trajectory of the CoM is planned by LIPM and LPM in the SSP and the DSP, respectively. According to the characteristics of LIPM and LPM, some geometric relations can ensure that acceleration of the CoM is continuous at the switch of SSP and DSP. Moreover, according to the regulation of the DSP, the biped robot can walk flexibly. The main work of this paper is as follows: Firstly, LIPM and LPM are introduced, their dynamic equations are analyzed, and their analytical solutions are given. Secondly, the walking stability of the robot during the SSP and DSP is analyzed. Then, several trajectory-planning methods are proposed under different situations. At last, simulations are carried out and simulation results show that the biped robot can walk stably and flexibly based on our proposed gait-planning method. This validates the effectiveness of the proposed method.

## 2. LIPM and LPM

To simplify the biped model, we consider the biped robot as a concentrated mass with massless legs. In this paper, we only consider the motion of the sagittal plane. LIPM and LPM are very simple and their dynamic equations have analytical solutions. We use LIPM to plan the CoM’s trajectory for the SSP and LPM for the DSP. In this section, the dynamic equations of LIPM and LPM and their analytical solutions are discussed.

### 2.1. LIPM

**Figure 1:**
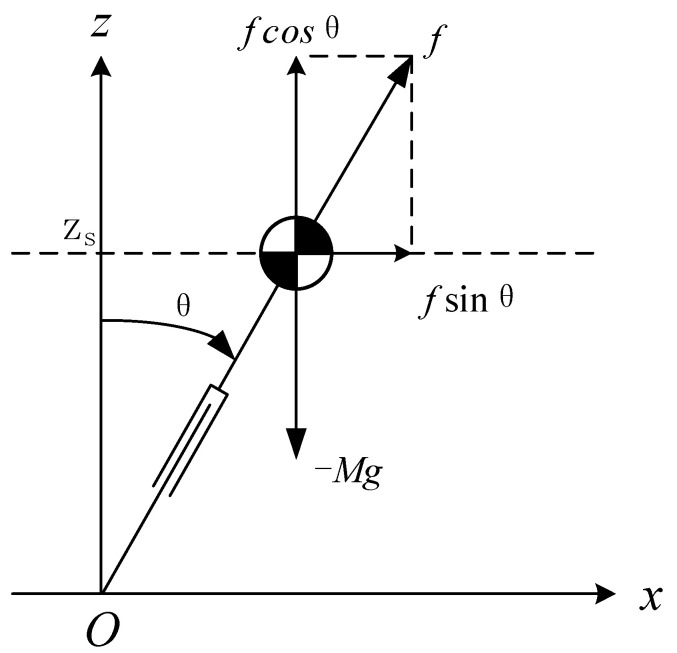
Linear inverted pendulum model-center of mass (LIPM-CoM) moving with constant height.

When the CoM of the robot moves along a horizontal straight line under the force of the massless leg, we call it a linear inverted pendulum, as shown in Figure 1. Readers interested can refer to Kajita et al. [27].

#### 2.1.1. Dynamic Equation with Constant Height of CoM

When the CoM moves along a horizontal straight line, its resultant force in the vertical direction is zero. Therefore, the dynamic differential equation of the CoM in the horizontal direction could be obtained by:

(1) $$\ddot{x}=\frac{g}{z_{s}}x$$

where $\left(x,z_{s}\right)$ is the position of the CoM. Because $z_{s}$ remains constant, the solution of the ordinary differential equation can be easily obtained by:

(2) $$x(t)=\frac{x(0)+T_{s}\dot{x}(0)}{2}e^{t/T_{s}}+\frac{x(0)-T_{s}\dot{x}(0)}{2}e^{-t/T_{s}}$$

(3) $$\dot{x}(t)=\frac{x(0)+T_{s}\dot{x}(0)}{2T_{s}}e^{t/T_{s}}+\frac{-x(0)+T_{s}\dot{x}(0)}{2T_{s}}e^{-t/T_{s}}$$

$$T_{s}\equiv \sqrt{\frac{z_{s}}{g}}$$

where $T_{s}$ is the time constant, which is related to the height of the CoM and the acceleration of gravity. $x\left(0\right),\dot{x}\left(0\right)$ is the initial state.

#### 2.1.2. Orbital Energy

Multiplying $\dot{x}$ on both side of the dynamic Equation (1), and integrating it, we can obtain:

(4) $$\frac{1}{2}{\dot{x}}^{2}-\frac{g}{2z_{s}}x^{2}=\mathrm{constant}\equiv E_{s}$$

The quantity $E_{s}$ is called orbital energy of LIPM. Equation (4) shows the conservation of orbital energy of LIPM.

#### 2.1.3. Transfer Time

In lots of situations, the time in which the CoM moves from one point to another is required. By doing some algebraic operations on Equations (2) and (3), we can get the following formula for calculating the transfer time:

(5) $$\tau_{s}=T_{s}\ln\frac{x_{1}+T_{s}{\dot{x}}_{1}}{x_{0}+T_{s}{\dot{x}}_{0}}$$

or:

(6) $$\tau_{s}=T_{s}\ln\frac{x_{0}-T_{s}{\dot{x}}_{0}}{x_{1}-T_{s}{\dot{x}}_{1}}$$

The results of Equations (5) and (6) are the same, unless one of them is singular by the numerator or denominator being zero.

#### 2.1.4. The CoM Moving along a Constrained Line

**Figure 2:**
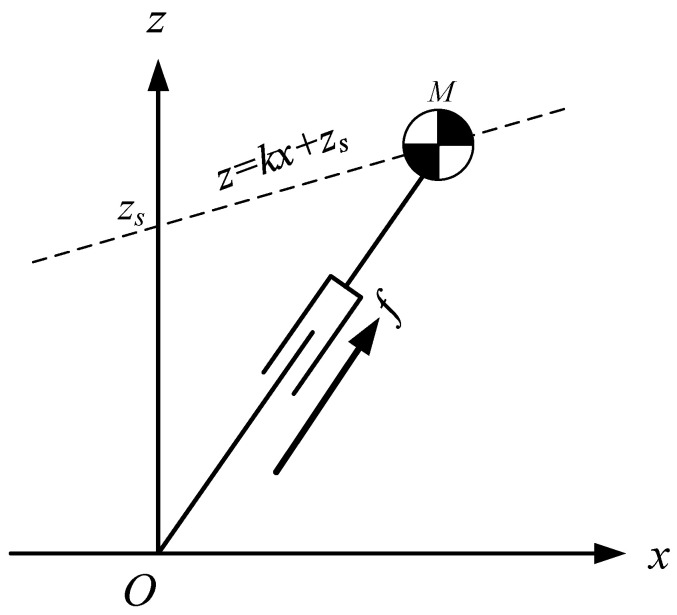
LIPM-CoM moving along a constraint line.

If the CoM moves along an oblique line, as shown in Figure 2, the equation of the oblique line is:

(7) $$z=k_{s}x+z_{s}$$

where $k_{s}$ is the slope of the line, $z_{s}$ is the intersection of the line and the Z-axis. This line is called the constraint line.

The dynamics of the CoM for LIPM in X-direction can be obtained by:

(8) $$\ddot{x}=\frac{g}{z_{s}}x$$

**Figure 3:**
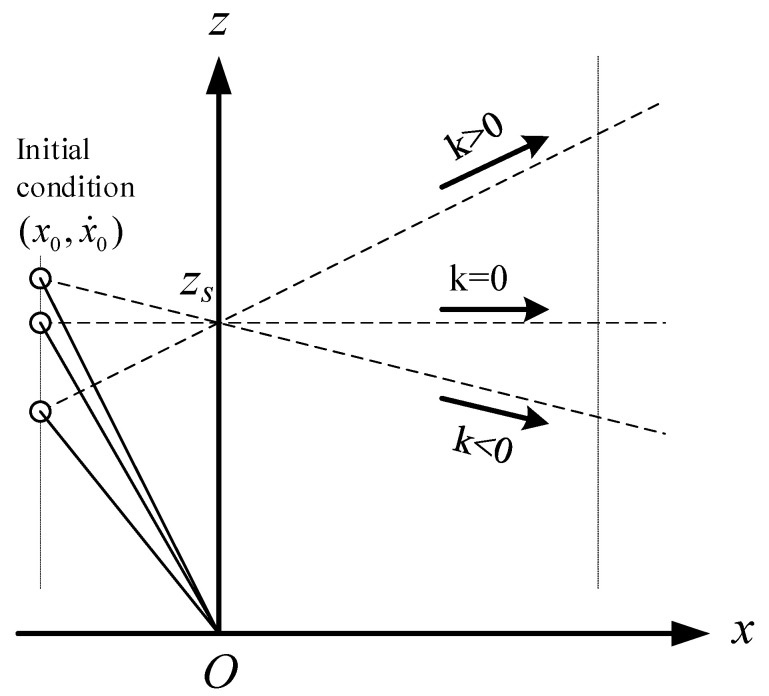
Motion of LIPM in X-direction is independent of the slope of the constraint line.

It is found that the equation of motion (8) is independent of the slope of the constraint line, and when CoM moves along the oblique line under the push force f, the dynamic Equation (8) is the same as the dynamic Equation (1) in X-direction. In other words, when the initial conditions in X-direction, $\left(x_{0},{\dot{x}}_{0}\right)$, are the same and the intersection points of constraint lines and Z-axis, $z_{s}$, are also the same, the three motions shown in Figure 3 are the same in X-direction.

### 2.2. LPM

**Figure 4:**
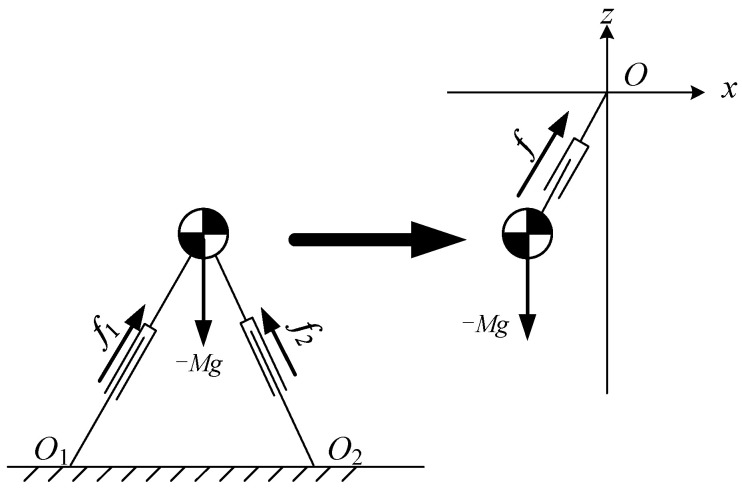
Pendulum model for the double support phase (DSP).

During the DSP, both feet of the robot are in contact with the ground. It can be considered that there are two kick forces applied to the CoM. Assume that two kick forces are $f_{1},f_{2}$, and their resultant force is *f*, as shown in Figure 4.

**Figure 5:**
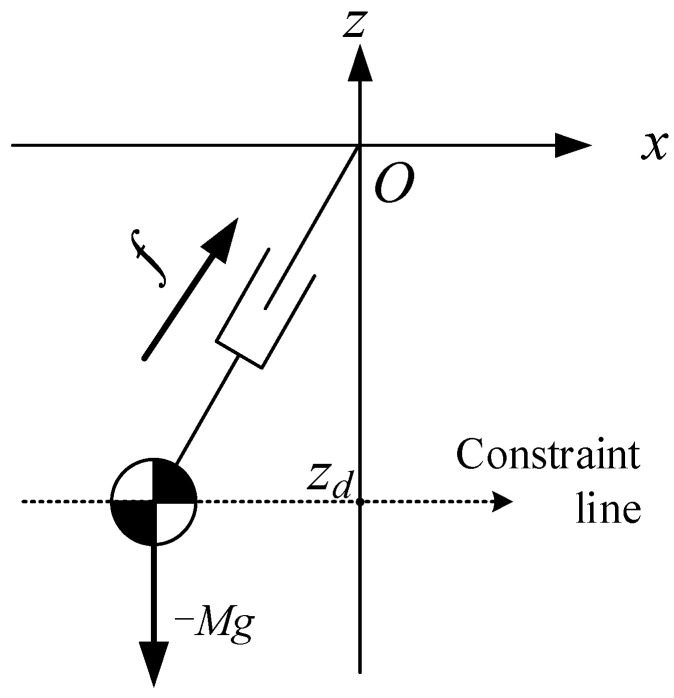
Linear pendulum model-center of mass (LPM-CoM) moving with a constant height.

Now suppose that there is a virtual suspending point above the CoM of the robot. A massless prismatic joint is used to connect the virtual suspension point and the CoM. The pull force of the prismatic joint on the CoM is equal to the force *f*. In this situation, the robot is modeled as a pendulum model. When the CoM of the pendulum moves along a constraint line, it becomes a Linear Pendulum Model, as shown in Figure 5.

#### 2.2.1. Dynamic Equation with a Constant Height of CoM

The local coordinate system is established with the virtual suspension point as the origin, and $\left(x,z_{d}\right)$ is the position of the CoM with respect to the virtual suspending point O. It is worth to note that $z_{d}$ is always negative. Similar to LIPM, the dynamic equation of the CoM is as follows:

(9) $$\ddot{x}=\frac{g}{z_{d}}x$$

It seems that Equation (9) is the same as Equation (1). However, we should note that $z_{s}>0,$ whereas $z_{d}<0$. To highlight $z_{d}<0$, Equation (9) can also be written as:

(10) $$\ddot{x}=-\frac{g}{\left|z_{d}\right|}x$$

The solution of the differential Equation (9) or (10) is:

(11) $$x(t)=x(0)\cos\left(\frac{t}{T_{d}}\right)+T_{d}\dot{x}(0)\sin\left(\frac{t}{T_{d}}\right)$$

(12) $$\dot{x}(t)=-\frac{1}{T_{d}}x(0)\sin\left(\frac{t}{T_{d}}\right)+\dot{x}(0)\cos\left(\frac{t}{T_{d}}\right)$$

$$T_{d}=\sqrt{\frac{\left|z_{d}\right|}{g}}$$

#### 2.2.2. Orbital Energy

Similar to LIPM, LPM also maintains the conservation of orbital energy, multiplying both sides of (10) by $\dot{x}$ and then integrating it, we can obtain:

(13) $$\frac{1}{2}{\dot{x}}^{2}+\frac{g}{2\left|z_{d}\right|}x^{2}=\mathrm{constant}\equiv E_{d}$$

The quantity $E_{d}$ is called the orbital energy of the LPM. Equation (13) shows the conservation of the LPM’s orbital energy of the LPM.

#### 2.2.3. Transfer Time

Sometimes, the time duration in which the CoM of the LPM moves from one point to another is required. Due to the periodic motion of the LPM, there are infinite solutions to the transfer time. We only want the time in which CoM reaches the final state for the first time. By doing some algebraic operations on (11) and (12), the transfer time can be obtained by:

(14) $$\tau_{d}=T_{d}\cos^{-1}\left(\frac{x_{0}x_{1}+T_{d}^{2}{\dot{x}}_{0}{\dot{x}}_{1}}{x_{0}^{2}+T_{d}^{2}{\dot{x}}_{0}^{2}}\right)$$

#### 2.2.4. The CoM Moving along a Constrained Line

**Figure 6:**
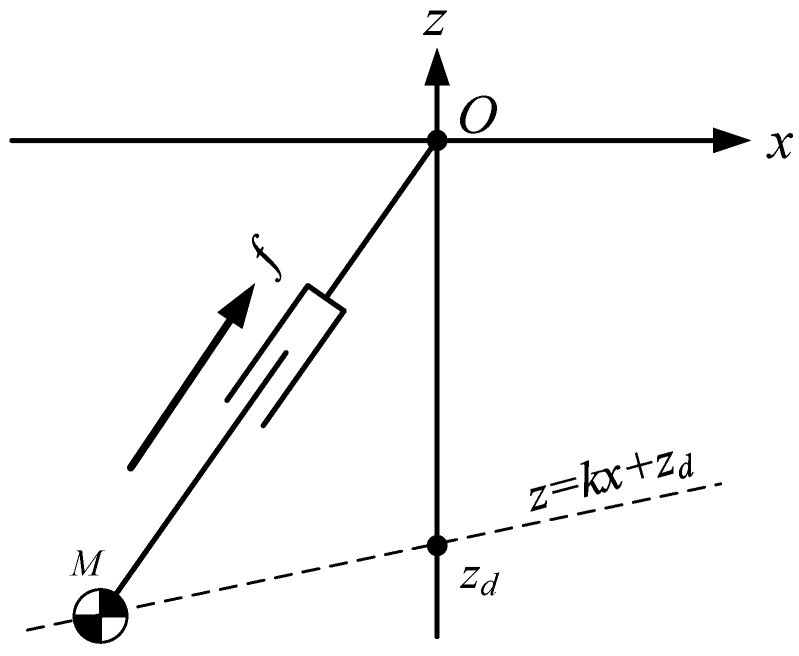
LPM-CoM moving along a constraint line.

If the CoM of the pendulum moves along an oblique line, as shown in Figure 6, the equation of the oblique line is:

(15) $$z=k_{d}x+z_{d},\;z_{d}<0$$

where $k_{d}$ is the slope of the line, $z_{d}$ is the intersection of the line and the Z-axis. This line is called the constraint line.

The dynamics of the CoM for LPM in X-direction can be obtained by:

(16) $$\ddot{x}=\frac{g}{z_{d}}x=-\frac{g}{\left|z_{d}\right|}x$$

**Figure 7:**
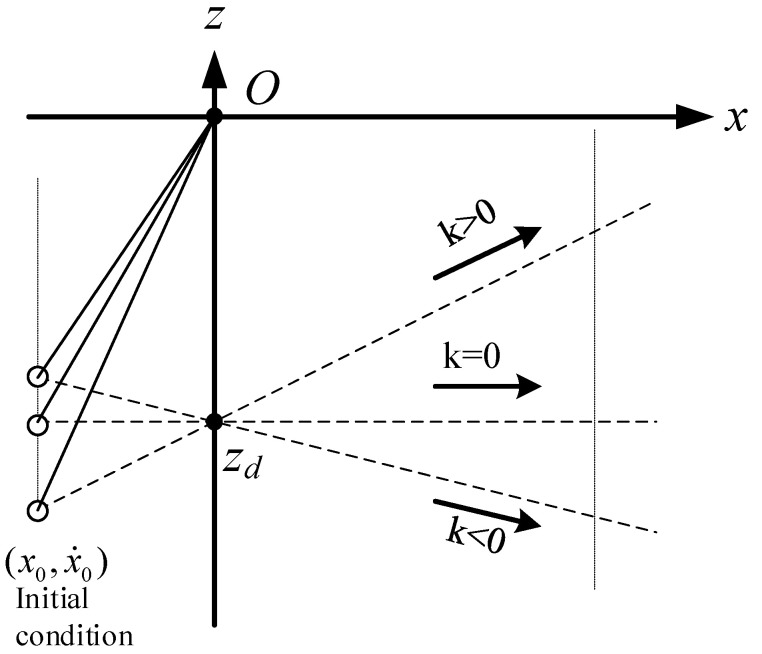
Motion of LPM in X-direction is independent of the slope of the constraint line.

It is found that the equation of motion (16) is also independent of the slope of the constraint line, and when the CoM moves along the constraint line under the pull force f, the dynamic Equation (16) is the same as the dynamic Equation (10) in the X-direction. In other words, when the initial conditions in X-direction, $\left(x_{0},{\dot{x}}_{0}\right)$, are the same and the intersection points of constraint lines and Z-axis are also the same, the three motions shown in Figure 7 are the same in X-direction.

## 3. Walking Stability

This section discusses the walking stability of LIPM and LPM. The ZMP stability criterion is chosen in this paper. When the ZMP/CoP remains in the support polygon, it can ensure that the robot’s feet will not turn over.

**Figure 8:**
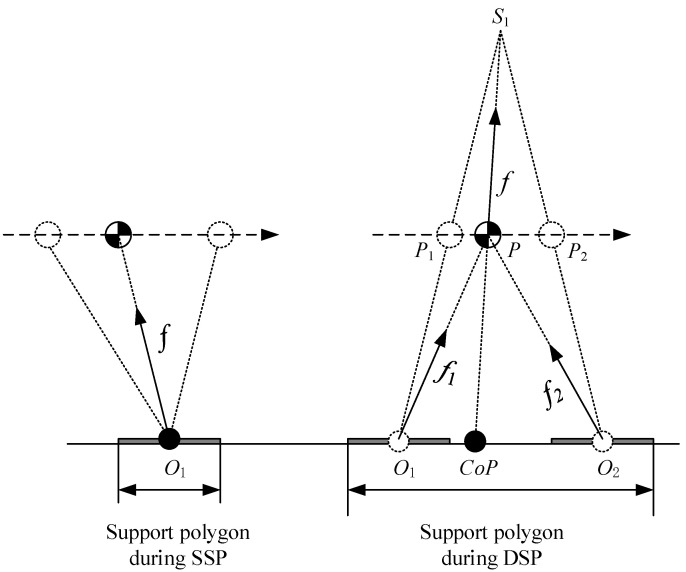
CoP during the SSP and DSP.

During the SSP, the CoM is pushed by prismatic joint. Since it is assumed that there is no torque at the pivot, CoP is the support point $O_{1}$ of LIPM, as shown in Figure 8.

During the DSP, two forces $f_{1}$ and $f_{2}$ are applied on CoM by two legs, and their resultant force is the pull force f of the prismatic joint in LPM. Suppose that the position of CoM is P at a certain moment in the DSP, as shown in Figure 8, then the CoP is the intersection point between the line $S_{1}P$ and the ground, where $S_{1}$ is the virtual suspending point of LPM. When the CoM gradually moves from $P_{1}$ to $P_{2}$, the CoP gradually moves from $O_{1}$ to $O_{2}$, where $O_{1}$ and $O_{2}$ are the support points of LIPM for the previous step and next step, respectively. At the beginning of the DSP, $f_{2}=0$ and $f_{1}=f$, this confirms an impactless landing of the swing foot; at the end of the DSP, $f_{1}=0$ and $f_{2}=f$, the rear foot of the robot can be naturally lifted off to switch the next SSP.

## 4. Trajectory-Planning Method

According to the LIPM and LPM introduced in Section 3, the trajectory of the CoM in the SSP and DSP can be planned respectively. In this section, several trajectory-planning methods are presented for periodic walking, disturbed state recovery, variable speed walking, and walking terrain-blind.

### 4.1. Continuity Condition of CoM Acceleration at the Transition between LIPM and LPM

If a certain geometric relationship is satisfied at the moment of the switch between the SSP and DSP, the acceleration of the CoM of LIPM and LPM at transition can be guaranteed to be equal.

**Figure 9:**
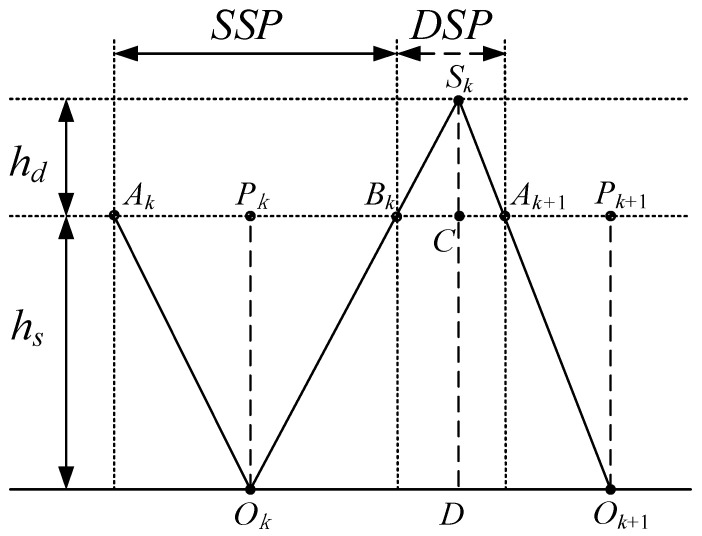
A schematic representation of LIPM and LPM.

Suppose the robot walks from left to right using LIPM in the SSP and LPM in the DSP, as shown in Figure 9. The support point of LIPM in the SSP of the *k*-th step is $O_{k}$, $S_{k}$ is the virtual suspending point of LPM in the DSP of the *k*-th step, and $O_{k+1}$ is the support point of LIPM in the SSP of the (*k*+1)-th step. When the CoM moves to $B_{k}$, it changes from the SSP to DSP; when the CoM moves to $A_{k+1}$, it changes from the DSP to the SSP of the next step. If three points, $O_{k}$, $B_{k,}$ and $S_{k}$ are in a straight line, according to the geometric relationship and Equations (1) and (10), the acceleration of CoM will be continuous at $B_{k}$. Similarly, if three points, $O_{k+1}$, $A_{k+1},$ and $S_{k}$ are in a straight line, the acceleration of CoM will be continuous at $A_{k+1}$. In the following trajectory-planning methods, the continuity condition of CoM acceleration at transition is satisfied by default.

### 4.2. Periodic Walking

**Proposition** **1.** *When the robot walks using LIPM and LPM in the SSP and DSP, respectively, the virtual suspension point of LPM and the support point of LIPM are symmetrical, i.e., $\left|O_{k}S_{k}\right|=\left|O_{k+1}S_{k}\right|$ or $\left|O_{k}D\right|=\left|DO_{k+1}\right|$ as shown in Figure 9*.

**Proof** **ofTheorem1.** Using the method of proof by contradiction. Suppose $\left|O_{k}D\right|\neq \left|DO_{k+1}\right|$, without loss of generality, let $\left|O_{k}D\right|>\left|DO_{k+1}\right|$. According to geometric relationship, we have $\left|A_{k}P_{k}\right|<\left|P_{k}B_{k}\right|$ and $\left|B_{k}C\right|=\left|CA_{k+1}\right|$. According to conservation of orbital energy of LIPM for the SSP, ${\dot{x}}_{A_{k}}<{\dot{x}}_{B_{k}}$ can be obtained. According to conservation of orbital energy of LPM for the DSP, ${\dot{x}}_{B_{k}}<{\dot{x}}_{A_{k+1}}$ can be obtained. Therefore, we obtain ${\dot{x}}_{A_{k}}<{\dot{x}}_{A_{k+1}}$. This contradicts the periodic condition ${\dot{x}}_{A_{k}}={\dot{x}}_{A_{k+1}}$. □

Once the step length is determined, the distances that the CoM travels in the SSP and DSP are related to the height parameters $h_{s}$ and $h_{d}$:

(17) $$\left|A_{k}B_{k}\right|=\frac{h_{s}}{h_{s}+h_{d}}L$$

(18) $$\left|B_{k}A_{k+1}\right|=\frac{h_{d}}{h_{s}+h_{d}}L$$

**Figure 10:**
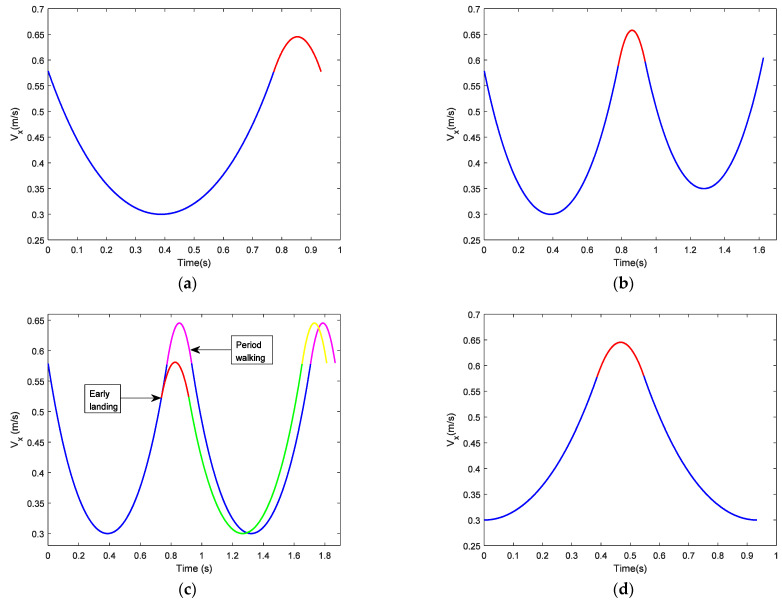
CoM velocities in X-direction under different conditions: (**a**) CoM velocity for period walking; (**b**) CoM velocity for adjusting walking speed with fixed step-length; (**c**) CoM velocity for unexpected landing time or foot placement; (**d**) CoM velocity walking on uneven ground.

The durations of the SSP and DSP are related to the walking speeds. Figure 10a shows the CoM velocity of period walking in X-direction. Height parameter of LIPM $h_{s}=0.9\;m$, height parameter of LPM $h_{d}=0.3\;m$, step length L = 0.4 m, CoM velocity of LIPM is 0.3 $m/s^{2}$ when it passes over the supporting point. In Figure 10a, the blue line is the one for the SSP, the red line is the one for the DSP. The time duration of the SSP is 0.77 s, the time duration of the DSP is 0.16 s. The average speed of period walking is 0.43 m/s.

### 4.3. Adjustment of Walking Speed with Fixed Step-Length

The walking speed of the biped can be adjusted by changing the touchdown time of the swing foot. Now we quantitatively analyze the touchdown timing of the swing foot when we adjust the robot’s walking speed by defining the orbital energies of LIPM of two adjacent steps as $E_{k}$ and $E_{k+1}$ respectively. Suppose that when the CoM passes through the point $P_{k}$, which is above support point $O_{k}$, its velocity is $v_{k}$; when the CoM reaches the point $B_{k}$, the DSP begins and the velocity is $v_{B}$; when the CoM reaches the point $A_{k+1}$, the next SSP begins, and the velocity is $v_{A}$; In the next SSP, when the CoM passes through the point $P_{k+1}$, which is above the support point $O_{k+1}$, its velocity is $v_{k+1}$, as shown in Figure 9. We have:

(19) $$E_{k}=\frac{1}{2}v_{k}^{2}=\frac{1}{2}v_{B}^{2}-\frac{g}{2h_{s}}{\left|P_{k}B_{k}\right|}^{2}$$

And:

(20) $$E_{k+1}=\frac{1}{2}v_{k+1}^{2}=\frac{1}{2}v_{A}^{2}-\frac{g}{2h_{s}}{\left|A_{k+1}P_{k+1}\right|}^{2}$$

According to the conservation of orbital energy of LPM during the DSP, the following equation holds:

(21) $$\frac{1}{2}v_{B}^{2}+\frac{g}{2h_{d}}{\left|B_{k}C\right|}^{2}=\frac{1}{2}v_{A}^{2}+\frac{g}{2h_{d}}{\left|CA_{k+1}\right|}^{2}$$

Substituting (19) and (20) into (21) yields:

(22) $$E_{k}+\frac{1}{2h_{s}}{\left|P_{k}B_{k}\right|}^{2}+\frac{g}{2h_{d}}{\left|B_{k}C\right|}^{2}=E_{k+1}+\frac{1}{2h_{s}}{\left|A_{k+1}P_{k+1}\right|}^{2}+\frac{g}{2h_{d}}{\left|CA_{k+1}\right|}^{2}$$

Let $L=\left|O_{k}O_{k+1}\right|$, $\alpha =\frac{\left|O_{k}D\right|}{\left|O_{k}O_{k+1}\right|}$ and $\beta =\frac{h_{d}}{h_{d}+h_{s}}$. According to the geometric relationship, we can get the following results:

(23) $$\{\begin{array}{l} B_{k}C=\beta \alpha L\\ P_{k}B_{k}=(1-\beta )\alpha L\\ CA_{k+1}=\beta (1-\alpha )L\\ A_{k+1}P_{k+1}=(1-\beta )(1-\alpha )L\\ h_{d}=\frac{\beta }{1-\beta }h_{s} \end{array}$$

Substituting (23) into (22) yields:

(24) $$\begin{array}{l} E_{k}+\frac{g}{2h_{s}}{\left(1-\beta \right)}^{2}\alpha^{2}L^{2}+\frac{g}{2h_{d}}\beta^{2}\alpha^{2}L^{2}\\ =E_{k+1}+\frac{g}{2h_{s}}{\left(1-\beta \right)}^{2}{\left(1-\alpha \right)}^{2}L^{2}+\frac{g}{2h_{d}}\beta^{2}{\left(1-\alpha \right)}^{2}L^{2} \end{array}$$

Let:

(25) $$\begin{array}{ll} \gamma & =\frac{g}{2h_{s}}{\left(1-\beta \right)}^{2}L^{2}+\frac{g}{2h_{d}}\beta^{2}L^{2}\\ & =\frac{g}{2h_{s}}{\left(1-\beta \right)}^{2}L^{2}+\frac{g(1-\beta )}{2h_{s}\beta }\beta^{2}L^{2}=\frac{g}{2h_{s}}(1+\beta )L^{2} \end{array}$$

Equation (25) can be simplified as:

(26) $$E_{k}+\gamma \alpha^{2}=E_{k+1}+\gamma {\left(1-\alpha \right)}^{2}$$

We can obtain:

(27) $$E_{k}-E_{k+1}=\gamma \left[{\left(1-\alpha \right)}^{2}-\alpha^{2}\right]=\gamma \left[1-2\alpha \right]$$

Then:

(28) $$\alpha =\frac{E_{k+1}-E_{k}}{2\gamma }+\frac{1}{2}=\frac{(E_{k+1}-E_{k})h_{s}}{g(1-\beta )L^{2}}+\frac{1}{2}$$

Once the step length L, and height parameters $h_{s}$ and $h_{d}$ are known, the position of the virtual suspension point $S_{k}$ can be determined. Suppose $O_{k}\left(0,0\right)$, then the coordinates of $S_{k}\left(x_{Sk},z_{Sk}\right),\;B_{k}\left(x_{Bk},z_{Bk}\right)$ and $A_{k+1}\left(x_{A_{k+1}},z_{A_{k+1}}\right)$ can be calculated as follows:

(29) $$\{\begin{array}{l} x_{S_{k}}=\alpha L\\ z_{S_{k}}=h_{s}+h_{d} \end{array}$$

(30) $$\{\begin{array}{l} x_{B_{k}}=(1-\beta )\alpha L\\ z_{B_{k}}=h_{s} \end{array}$$

(31) $$\{\begin{array}{l} x_{A_{k+1}}=L-(1-\alpha )(1-\beta )L\\ z_{A_{k+1}}=h_{s} \end{array}$$

Figure 10b shows the adjustment of walking speed with fixed step-length. Height parameter of LIPM $h_{s}=0.9\;m$, height parameter of LPM $h_{d}=0.3\;m$, step length *L* = 0.4 m. The current step begins at the beginning of the SSP for period walking with the apex velocity 0.3 m/s, i.e., the velocity at which the CoM passes over the supporting point of LIPM. Through the adjustment of the DSP, apex velocity of the SSP in the next step becomes 0.35 m/s. In Figure 10b the blue line is the CoM’s velocity during the SSP, and the red line is the CoM’s velocity during the DSP.

### 4.4. Trajectory Planning for Unexpected Landing Time or Foot Placement

**Figure 11:**
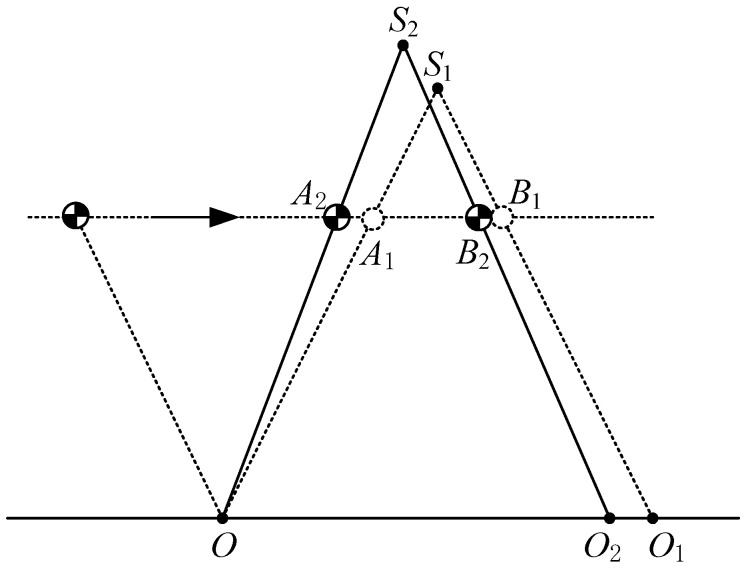
Trajectory planning for unexpected landing time or foot placement.

Sometimes, the swing foot lands aground at an unexpected time or in an unexpected foot placement. In these situations, the CoM enters the DSP with an unexpected state. Trajectory of the CoM during the DSP should be adjusted in real time to retain the orbital energy in the next SSP. Figure 11 shows one of these situations, i.e., the swing foot lands on the ground early and with a smaller step length. Under the previous plan, the swing foot lands on the point $O_{1}$ when the CoM reaches the point $A_{1}$; the CoM moves from $A_{1}$ to $B_{1}$ during the DSP; the virtual suspending point of LPM is at the point $S_{1}$. Now the biped lands on the point $O_{2}$ when the CoM reaches the point $A_{2}$. We have to replan the trajectory of the CoM during the DSP to ensure the same orbital energy of the next SSP. To this end, the main problem is to identify the position of the new virtual suspending point. Point O and $A_{2}$ define a line and we can know the slope of this line. The next point $O_{2}$ and the same slope of the previous line only with a negative sign define a new line. The intersection of two lines, $S_{1}$, is the new virtual suspending point. The intersection of the second line and the constant height line, $B_{2}$, is the new position at which the DSP ends.

Figure 10c shows trajectory planning for unexpected landing time or unexpected foot placement. Suppose a robot is walking with a period gait, which is also shown in Figure 10c. The parameters for period walking are *L* = 0.4 m, the apex velocity of LIPM is 0.3 m/s, $h_{s}=0.9$ m, $h_{d}=0.3$ m. In period walking, CoM should reach the point $A_{1}$ when the swing foot lands the ground on the point $O_{1}$ as illustrated in Figure 11. For some reason, when CoM moves to point $A_{2}$, the swinging foot lands the ground on point $O_{2}$. Suppose the origin of the local coordinate frame is at the support point O, $x_{A2}=0.13$ m, $x_{O2}=0.36$ m (see Figure 11). Due to the early landing of the swinging foot, it is necessary to adjust the CoM state in the DSP to make it return to the periodic gait in the next SSP. In Figure 10c, the blue line (for the SSP) and the pink line (for the DSP) is the CoM velocity of period walking, the red line is the adjustment of the DSP for early landing, and the green line and yellow line are the CoM velocity of the next step after adjustment. It is observed that after adjustment, the CoM state returns to the period gait.

Suppose O(0,0) and CoM reaches $A_{2}\left(x_{A2},h_{s}\right)$ when the swing foot lands on the ground at $O_{2}\left(x_{O2},0\right)$, then we can calculate the coordinate of $S_{2}\left(x_{S2},z_{S2}\right)$ and $B_{2}\left(x_{B2},h_{s}\right)$ as follows:

(32) $$\{\begin{array}{l} x_{S2}=\frac{x_{O2}}{2}\\ z_{S2}=\frac{z_{A2}x_{O2}}{2x_{A2}} \end{array}$$

(33) $$x_{B2}=x_{O2}-\frac{x_{A2}}{z_{A2}}h_{s}$$

### 4.5. Realtime Trajectory Planning for Terrain-Blind Walking

**Figure 12:**
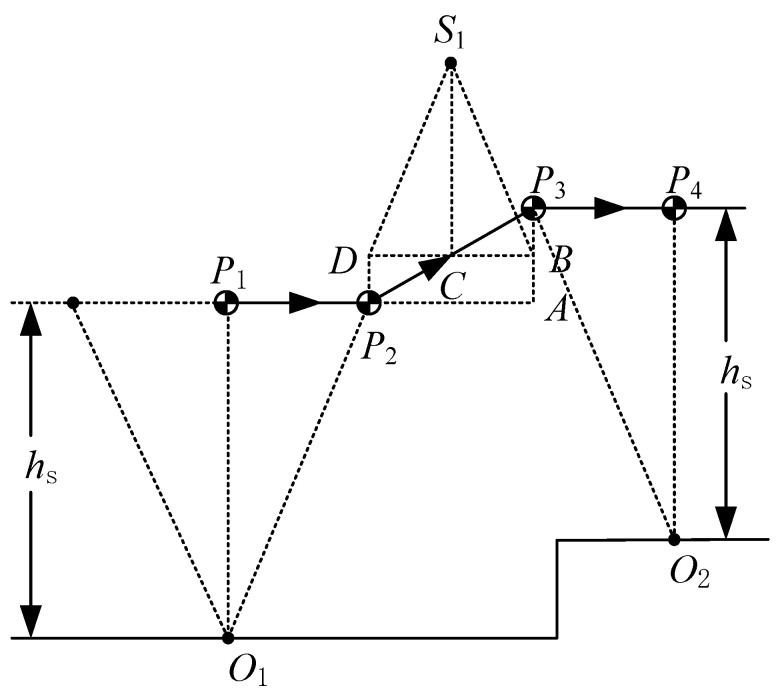
Realtime trajectory planning for terrain-blind walking.

When a biped robot walks from laboratory to the outdoor environment, the walking surface is not flat any more. If the biped is not equipped with a visual sensing system, it cannot obtain the height of a new landing point predictively. However, the height of the new landing point can be obtained by the joint sensor at the moment of the robot’s foot landing on the ground. In this situation, we also need to replan the trajectory of the CoM during the DSP. As shown in Figure 12, the CoM reaches the point $P_{2}$ at the moment of the swing foot landing on point $O_{2}$. To make the figure clearer, we enlarged the height difference between two supporting points, $O_{1}$ and $O_{2}$. Next, we introduce how to determine the position of the suspending point.

Firstly, determine a point, $P_{4}$, over the support point $O_{2}$, to make $\left|O_{2}P_{4}\right|=h_{s}$. Secondly, determine a point, $P_{3}$, which satisfies the height of $P_{3}$ with respect to $O_{2}$ being $h_{s}$ and $k_{O_{2}P_{3}}=-k_{O_{1}P_{2}}$. Here $k_{*}$ is the slope of the specified line. Thirdly, determine point *A*, which is under $P_{3}$ and keeps a height $h_{s}$ with respect to $O_{1}$. Point B is the midpoint of point $P_{3}$ and point *A*. Point *D* is over $P_{2}$ and has the same height as point B. Finally, determine the virtual suspending point $S_{1}$, which satisfies $k_{S_{1}D}=k_{O_{1}P_{2}}$ and $k_{S_{1}B}=k_{O_{2}P_{3}}$. If the CoM of LPM moves from point *D* to point B, it is obvious that velocities at point *D* and B are the same in X-direction. As discussed in Section 2.2, CoM motion from $P_{2}$ to $P_{3}$ is the same as that from point *D* to point B in X-direction. Therefore ${\dot{x}}_{P_{2}}={\dot{x}}_{P_{3}}$, $\left|P_{1}P_{2}\right|=\left|P_{3}P_{4}\right|$ and $\left|O_{1}P_{1}\right|=\left|O_{2}P_{4}\right|$ ensure that the motion of the next LIPM is the same as that of the previous LIPM. As a result the position of $S_{1}$ in X-direction is at the center of points $O_{1}$ and $O_{2}$ in X-direction.

Suppose $O_{1}\left(0,0\right)$ and CoM reaches $P_{2}\left(x_{P2},z_{P2}\right)$ when the swing foot lands on the ground at $O_{2}\left(x_{O2},z_{O2}\right)$, then we can calculate the coordinate of $S_{1}\left(x_{S1},z_{S1}\right)$ and $P_{3}\left(x_{P3},z_{P3}\right)$ as follows:

(34) $$\{\begin{array}{l} x_{S1}=\frac{x_{O2}}{2}\\ z_{S1}=\frac{z_{O2}}{2}+\frac{x_{O2}z_{P2}}{2x_{P2}} \end{array}$$

(35) $$\{\begin{array}{c} x_{P3}=x_{O2}-x_{P2}\\ z_{P3}=z_{O2}+z_{P2} \end{array}$$

When the biped walks on uneven ground, its motion in X-direction is the same as the one walking on flat ground. The difference is the motion in Z-direction. For example, if we suppose the biped robot walks on uneven ground as illustrated in Figure 12, the height difference between points $O_{2}$ and $O_{1}$ is 0.04 m, step length is 0.4 m, other parameters are $h_{s}=0.9$ m, $\left|P_{1}P_{2}\right|=0.15$ m, and the apex velocity of LIPM is 0.3 m/s. We chose these parameters to be the same as period walking deliberately. Figure 10d shows the CoM velocity in X-direction from point $P_{1}$ to point $P_{4}$ through points $P_{2}$ and $P_{3}$. This is the same as the period walking of Figure 10a.

### 4.6. Flow Chart of Proposed Methods

**Figure 13:**
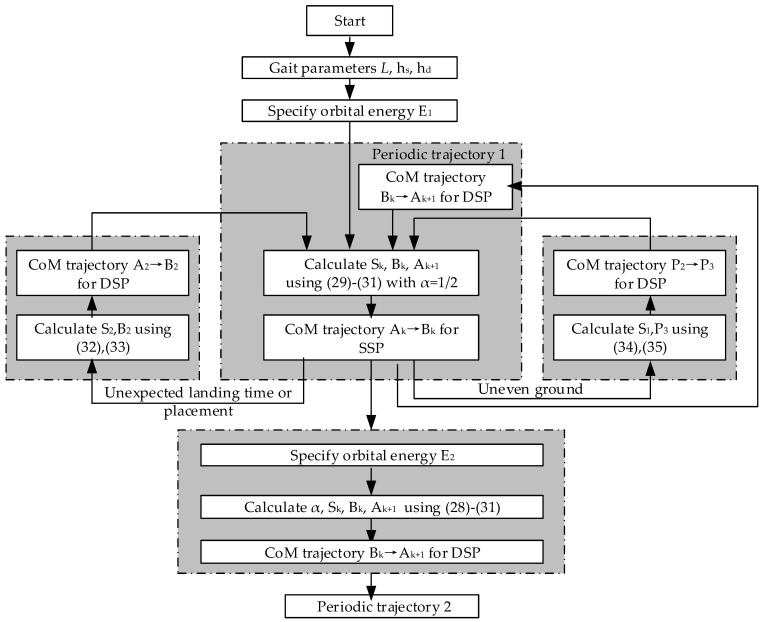
Flow chart of proposed methods including periodic walking, adjusting walking speed, recovery of unexpected landing, and walking on uneven ground. In the blocks of periodic walking and adjusting walking speed, A_k_, B_k_, S_k_ and A_k+1_ are shown in Figure 9. In the block of unexpected landing time or placement, A_2_, B_2_ and S_2_ are shown in Figure 11. In the block of uneven ground, P_2_, P_3_ and S_1_ are shown in Figure 12.

In this section, we propose several trajectory-planning methods for the CoM, i.e., periodic walking, adjusting walking speed, recovery of unexpected landing, and walking on uneven ground. The flowchart of overall idea is shown in Figure 13.

## 5. Simulation

In this section, simulation results of the proposed trajectory-planning method using LIPM and LPM are illustrated. To demonstrate the implementation of the research, some simulations are produced in a physical scenario.

### 5.1. Modeling Reality

**Figure 14:**
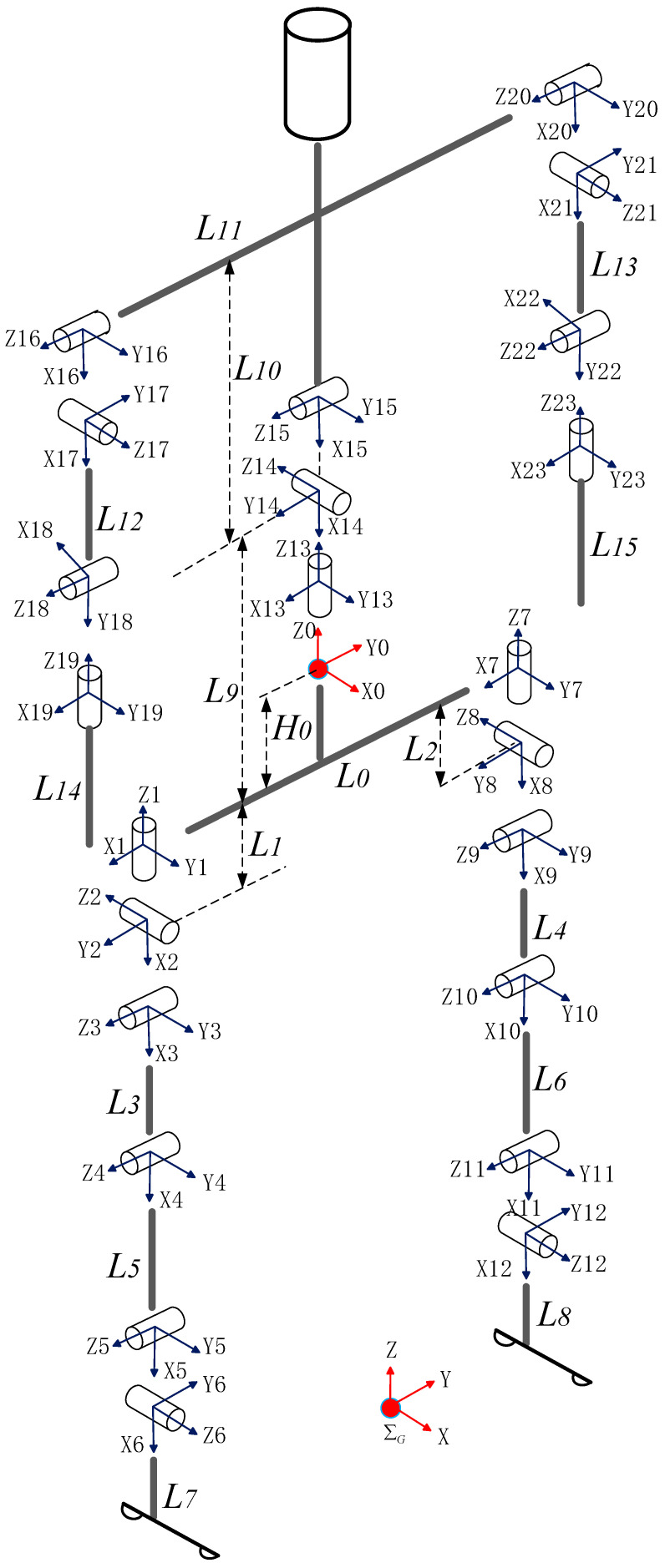
Kinematic modelling.

We develop the simulation platform in Matlab Simscape. The simulation model is built according to the prototype developed by our laboratory. The robot’s height is 1.34 m, and its total mass is about 40 kg. It has 23 degrees of freedom. The structure of the biped is shown in Figure 14 and the detail physical parameters is listed in Table 1. The humanoid robot is treated as a tree-structure topology with a floating base. Here, we set the pelvis as the floating base and number it link 0, as shown in Figure 14. Herein, we will fix some degrees of freedom as needed. 

**Figure 15:**
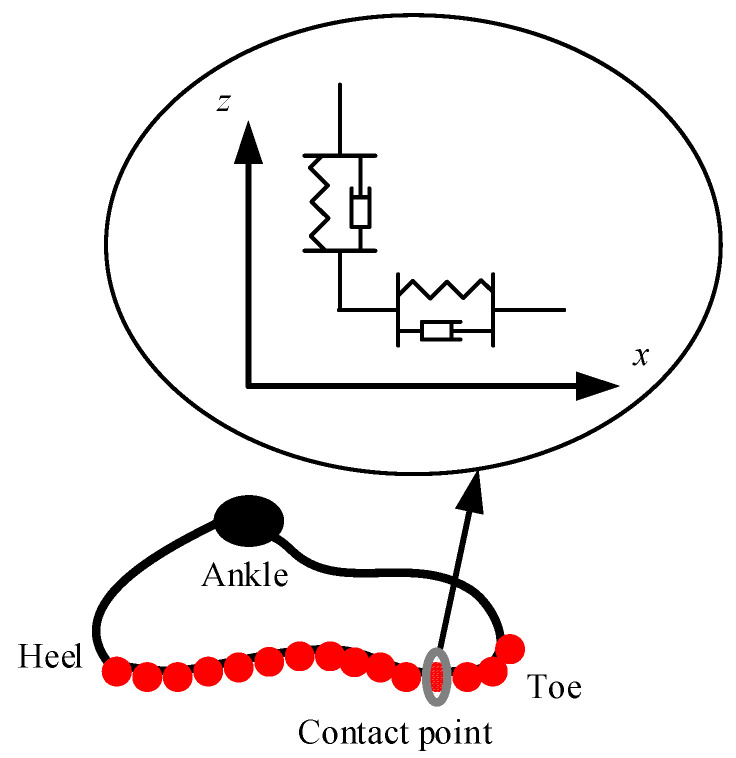
Contact model of the foot and ground.

In this paper, the contact model in the normal direction between the feet and ground is assumed to be a distributed and nonlinear spring-damper model [34]. A number of contact points are located under the sole like the structure of the foot shoe, as shown in Figure 15. Each contact point is composed of two nonlinear springs and two nonlinear dampers connected in parallel pattern. The contact force can be expressed as follows:

(36) $$\{\begin{array}{l} f_{x}=-k_{x}\Delta x{\left|\Delta z\right|}^{\rho }-c_{x}\Delta \dot{x}{\left|\Delta z\right|}^{\zeta }\\ f_{z}=\{\begin{array}{ll} k_{z}{\left|\Delta z\right|}^{\rho }, & \Delta z\leq 0\;\mathrm{and}\;\Delta \dot{z}\geq 0\\ k_{z}{\left|\Delta z\right|}^{\rho }+c_{z}\Delta \dot{z}{\left|\Delta z\right|}^{\zeta }, & \Delta z\leq 0\;\mathrm{and}\;\Delta \dot{z}\leq 0\\ 0, & \Delta z>0 \end{array} \end{array}$$

Here, (x,z) is the current position of the contact point in the world coordinate system; $\left(x_{\mathrm{grd}},z_{\mathrm{grd}}\right)$ is the initial position of the contact point; $\Delta x=x-x_{\mathrm{grd}}$ denotes the tangential offset of the deformation; $k_{x}$ and $c_{x}$ are the stiffness coefficient and damper coefficient in the tangential direction, respectively; $\Delta z=z-z_{\mathrm{grd}}$ denotes the penetration depth of the ground; $k_{z}$ and $c_{z}$ are the stiffness coefficient and damper coefficient in the normal direction, respectively; ρ and ζ describe the influences of the penetration depth on the stiffness and damper coefficients—these two parameters have distinct physical meanings: the greater the penetration depth, the closer the contact of body surfaces, and the more the stiffness and the damper weight. Experimentally, the parameters of the contact model are shown in Table 2.

In the simulation platform, a proportion-integration-differentiation (PID) controller is used for each joint. PID parameters of each joint are listed in Table 3.

### 5.2. Desired Trajectories in Simulations

In Section 4, we only planned the trajectories of CoM during the SSP and DSP using LIPM and LPM, respectively. In actual robot walking, we need to control the movement of each joint, so we need to know the desired angle trajectory of each joint. In this paper, we only control the lower limbs of the robot and do not consider movements of the upper limbs, such as two arms and head. Because the weight of the robot legs is relatively small, we set the total CoM of the robot at the CoM of the trunk. Hence the position of the hip joint can be calculated according to the CoM position. The robot keeps its trunk upright and its feet parallel to the ground during walking. If we know the trajectories of the hip and two ankles, we can obtain the angle trajectories of hip joint, knee joint, and ankle joint using inverse kinematics.

In order to reduce the adverse impact of collision, the velocity of the swing ankle is designed to be zero when landing. In simulations, the desired trajectory of the swing ankle is as follows:

(37) $$x_{\mathrm{swing}}=x_{\mathrm{stance}}-2(S_{1}+S_{2})\tau^{3}+3(S_{1}+S_{2})\tau^{2}-S_{1}$$

(38) $$z_{\mathrm{swing}}=R(1-\cos(2\pi \tau ))$$

Here, $S_{1}$ is the horizontal distance between stance ankle and swing ankle when swing foot takes off from the ground, and $S_{2}$ is the horizontal distance between stance ankle and swing ankle when swing foot lands on the ground; $\tau =t/T_{ss},t\in \left[0,T_{ss}\right]$ and $T_{ss}$ is the duration of the SSP; R is the height parameter of the swing ankle.

### 5.3. Walking on Level Ground

**Figure 16:**
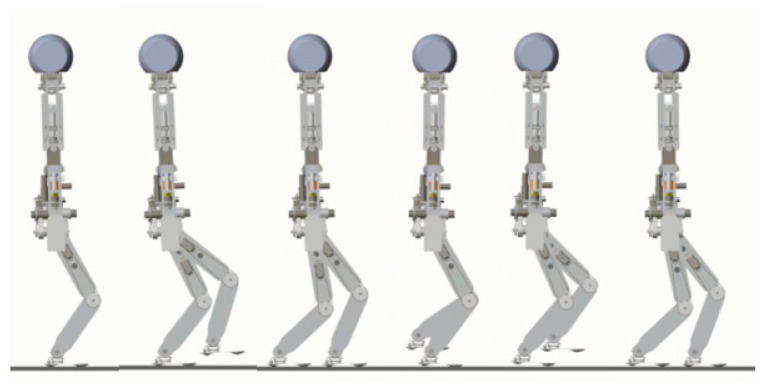
Snapshots of the first simulation: the biped walks on level ground.

In the first simulation, the biped starts to walk from standing still on a level ground. The parameters for gait generation are: *L* = 0.25 m, $h_{s}=0.85$ m, $h_{d}=0.3$ m. The biped stands still at the beginning. At the moment *t* = 1.0 s, it starts to walk and increase the walking speed gradually in the next three steps. From the fourth step, it starts cyclic walking. Then at the seventh step, its foot lands on the ground early with a step length of 0.2 m. Then it returns to the cyclic gait in the next step and goes ahead until the eleventh step. Figure 16 shows the snapshots of the simulation.

**Figure 17:**
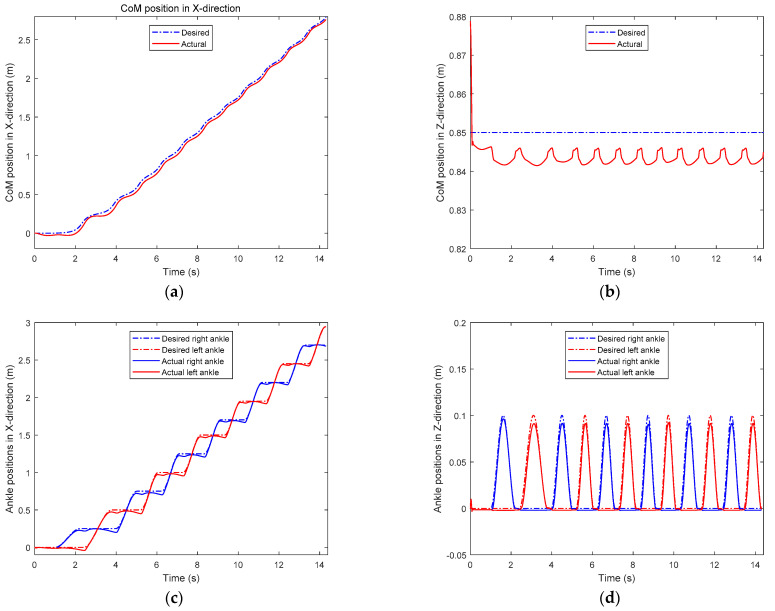
The difference between desired and actual trajectories: (**a**) CoM positions in X-direction; (**b**) CoM positions in Z-direction; (**c**) ankle positions in X-direction; (**d**) ankle positions in Z-direction.

Figure 17 shows the desired trajectories of CoM and two ankles in X- and Z-directions, respectively. We can observe from Figure 17a that the actual CoM position is behind the desired one. This is because of the control error. From Figure 17b, we can observe that the actual CoM height is slightly lower than the desired one. This is mainly caused by the control error, for example, the stance of the knee is more bent than desired, and there is foot subsidence with the nonlinear spring-damper ground. In addition, there is a decrease in the actual CoM height at the beginning of the simulation. This is because the biped starts the simulation with two legs parallel and straight, it needs to bend its knees to adjust the CoM. Ankle positions are similar to CoM positions. Due to the gravity and control error, the height of the swing ankle is lower than desired—swing foot lands on the ground earlier than the expected time.

**Figure 18:**
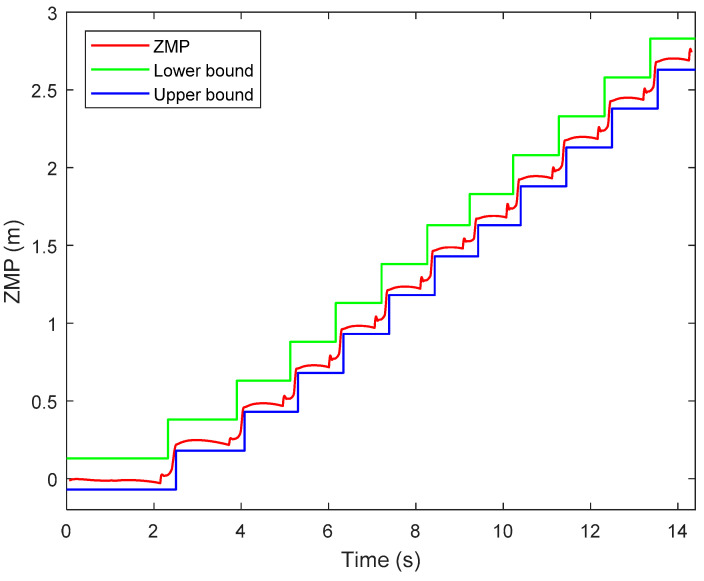
Zero-moment point (ZMP) trajectory in the first simulation.

**Figure 19:**
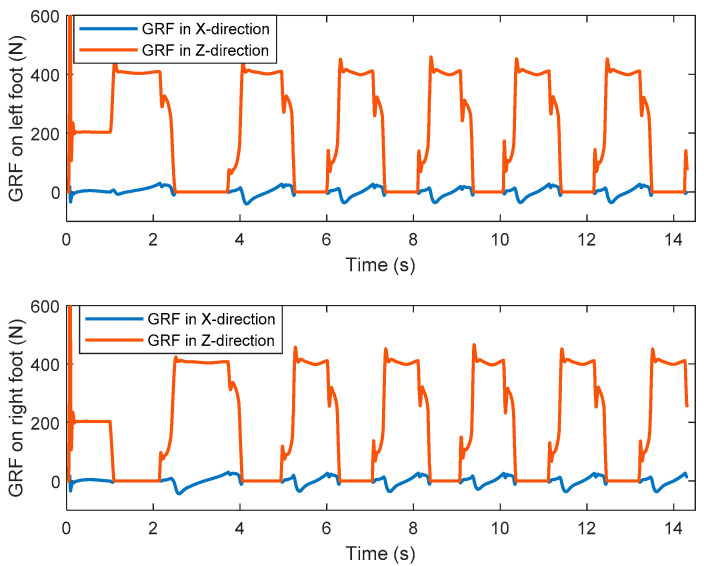
The ground reaction forces of the left and right feet in the first simulation.

Figure 18 shows the ZMP trajectory in the first simulation. It can be observed that the ZMP stays in the ZMP region. In the simulation, the biped robot walks stably without falling down. Figure 19 shows the ground reaction force (GRF) during the whole simulation. It is noted that the GRF has very large magnitude at the beginning of the simulation. This is because our model assumes that the robot is suspended with zero height before the simulation, and there is a sudden gravity effect between the foot and the ground when the simulation starts. It is observed that the tangential component of the GRF is much smaller than the normal component of the GRF. It ensures no sliding between the foot and the ground.

### 5.4. Walking on Uneven Ground

**Figure 20:**
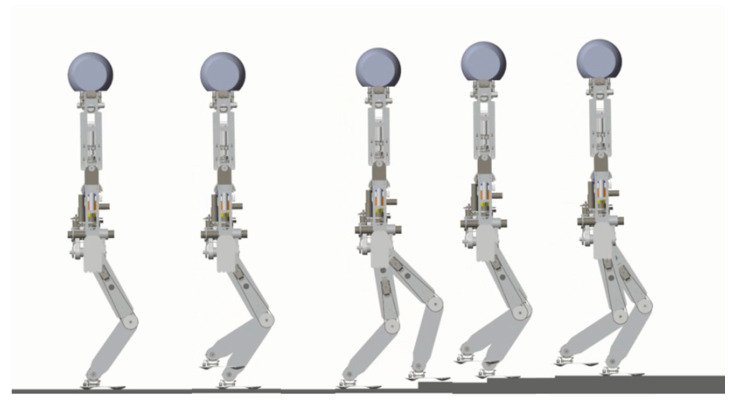
Snapshots of the second simulation: the biped walks on uneven ground.

The second simulation is to verify the terrain-blind walking ability using the proposed method. Similar to the first simulation, the biped robot stands still at the beginning, then speeds up to a cyclic walking gait. From the fifth to the eighth step, the ground is no longer flat. The height differences between adjacent two feet locations are 0.03 m, 0.02 m, 0.01 m, and 0.01 m, respectively. Figure 20 shows the snapshots of the second simulation.

**Figure 21:**
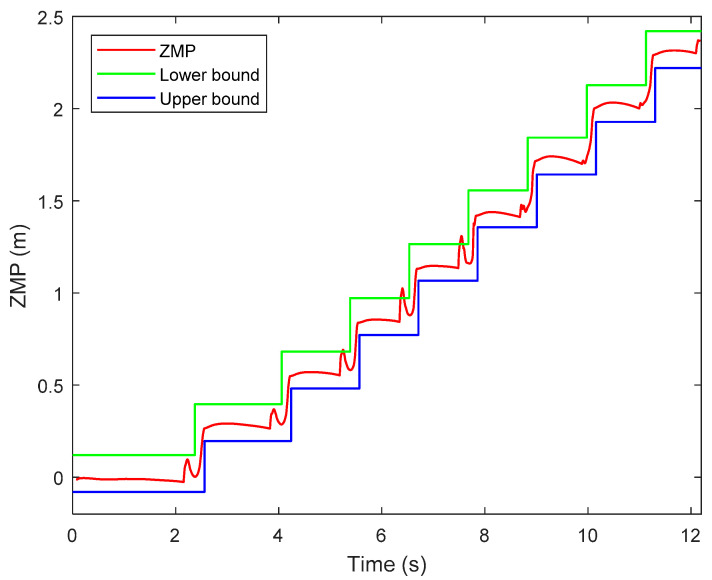
ZMP trajectory in the second simulation.

**Figure 22:**
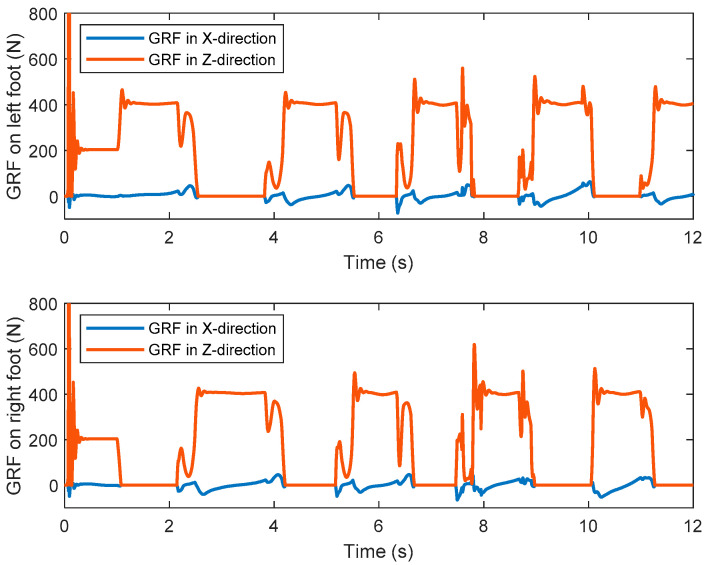
The ground reaction forces of the left and right feet in the second simulation.

Similar to the first simulation, Figure 21 shows the ZMP trajectory in the second simulation and Figure 22 shows the GRF in the second simulation. From Figure 21, the ZMP measured by the contact model almost exits within the desired support polygon. Hence, stable walking is guaranteed. At some moments, the ZMP escapes from the stable region, which may be caused by numerical and control errors. The ZMP margin is the analytic value computed in the stage of trajectory planning. In simulation, however, the swing ankle position is lower than the desired one, caused by control error, which makes the swing foot touch the ground a little earlier than the desired moment. The unexpected touchdown of the swing foot generates an impact between the foot and the ground. Therefore, the actual ZMP is out of the ZMP margin temporarily at the moment of touchdown. Nevertheless, the biped robot will not lose its stability if the ZMP exceeds the stable region in a very short time. This situation can also be seen in other references, such as [35,36,37]. From Figure 22, it is observed that the biped can walk stably with no sliding.

## 6. Conclusions

In this paper, we proposed a trajectory-planning method that used LIPM for the SSP and LPM for the DSP. The dynamic equations of these two models have analytical solutions, so it is very easy to plan the CoM trajectory. The walking stability of LIPM and LPM is also discussed. Through dynamics analysis, walking stability of a planned trajectory can be ensured.

Using the proposed method, the trajectory of a biped robot can be generated in real time. Some trajectory-planning methods are presented. Periodic trajectory can be generated if some walking parameters are specified, such as step length, height parameters for LIPM and LPM, and apex velocity. We can also change the walking speed of the robot with a fixed step-length by adjusting the virtual suspending point of LPM. Disturbed by interference, the swing leg of the biped maybe lands at an unexpected time or on an unexpected foot placement. Through the adjustment of the DSP, the state of the biped can return to its original planned trajectory. We also present a trajectory-planning method for walking on uneven ground with unknown height. Its horizontal motion is similar to the situation of flat ground with a different motion in the vertical direction. At last, simulations are carried out. In both simulations, the biped robot can walk stably using our proposed method. Simulations verify the effectiveness of the proposed method.

## Figures and Tables

**Table 1:** Physical parameters of the humanoid robot.

Link	*L*_0_	*L*_1_ *, L*_2_	*L*_3_ *, L*_4_	*L*_5_ *, L*_6_	*L*_7_ *, L*_8_	*L*_9_	*L*_10_	*L*_11_	*L*_12_, *L*_13_	*L*_14_ *, L*_15_	*H*_0_
Length/m	0.2	0.133	0.35	0.282	0.051	0.133	0.538	0.447	0.31	0.295	0.009
Mass/kg	1.862	4.025	3.305	1.385	0.236	5.019	8.907	0.312	2.113	1.334	

**Table 2:** Main parameters of the contact model.

$k_{x}$	$c_{x}$	$k_{z}$	$c_{z}$	ρ	ς
4.6 × 10^4^	9.0 × 10^5^	9.1 × 10^4^	2.0 × 10^6^	1.5	0.35

**Table 3:** PID parameters for each joint.

Control Parameters	K_P_	K_I_	K_D_
Hip joint	1500	0.1	40
Knee joint	2000	0.1	50
Ankle joint	800	0.1	40

## Data Availability

No new data were created or analyzed in this study. Data sharing is not applicable to this article.

## References

[1] Tzafestas S., Raibert M. (1996). Robust Sliding-mode Control Applied to a 5-Link Biped Robot. J. Intell. Robot. Syst..

[2] Vukobratovic M., Borovac B. (2004). Zero-Moment Point-Thirty five years of its life. Int. J. Hum. Robot..

[3] Goswami A. (1999). Postural stability of biped robots and the foot-rotation indicator (FRI) point. Int. J. Robot. Res..

[4] McGeer T. (1990). Passive dynamic walking. Int. J. Robot. Res..

[5] Collins S.H., Wisse M., Ruina A. (2001). A three-dimensional passive dynamic walking robot with two legs and knees. Int. J. Robot. Res..

[6] Freidovich L.B., Mettin U., Shiriaev A., Spong M.W. (2009). A passive 2-dof walker: Hunting for gaits using virtual holonomic constraInts. IEEE Trans. Robot..

[7] Grizzle J.W., Chevallereau C., Sinnet R.W., Ames A.D. (2014). Models, feedback control, and open problems of 3D bipedal robot walking. Automatica.

[8] Koolen T., De Boer T., Rebula J., Goswami A., Pratt J. (2012). Capturability-based analysis and control of legged locomotion, Part 1: Theory and application to three simple gait models. Int. J. Robot. Res..

[9] Shiriaev A.S., Freidovich L.B., Spong M.W. (2014). Controlled invariants and trajectory planning for underactuated mechanical systems. IEEE Trans. Automat. Control.

[10] Bessonnet G., Chesse S., Sardain P. (2004). Optimal gait synthesis of a seven-link planar biped. Int. J. Robot. Res..

[11] Bessonnet G., Chesse S., Sardain P. (2005). A parametric optimization approach to walking pattern synthesis. Int. J. Robot. Res..

[12] Tlalolini D., Aoustin Y., Chevallereau C. (2010). Design of a walking cyclic gait with single support phases and impacts for the locomotor system of a thirteen-link 3D biped using the parametric optimization. Multibody Syst. Dyn..

[13] Grizzle J.W., Abba G., Plestan F. (2001). Asymptotically stable walking for biped robots: Analysis via systems with impulse effects. IEEE Trans. Automat. Control.

[14] Buss B.G., Hamed K.A., Griffin B.A., Grizzle J.W. Experimental results for 3D bipedal robot walking based on systematic optimization of virtual onstraints. Proceedings of the 2016 American Control Conference(ACC).

[15] Farzadpour F., Danesh M., Torklarki S.M. (2015). Development of multi-phase dynamic equations for a seven-link biped robot with improved foot rotation in the double support phase. Proc. Inst. Mech. Eng. C J. Mech..

[16] Fakhari A., Fattah A. Trajectory Planning of Walking with Different Step Lengths of a Seven-Link Biped Robot. Proceedings of the ASME International Design Engineering Technical Conferences & Computers and Information in Engineering Conference.

[17] Paparisabet M.A., Dehghani R., Ahmadi A.R. (2019). Knee and torso kinematics in generation of optimum gait pattern based on human-like motion for a seven-link biped robot. Multibody Syst. Dyn..

[18] Dau V.H., Chew C.M., Poo A.N. Optimal trajectory generation for bipedal robots. Proceedings of the IEEE-RAS International Conference on Humanoid Robots.

[19] Rabah M., Rohan A., Kim S.H. (2018). Comparison of Position Control of a Gyroscopic Inverted Pendulum Using PID, Fuzzy Logic and Fuzzy PID controllers. Int. J. Fuzzy Log. Intell. Syst..

[20] Rohan A., Rabah M., Nam K.H., Kim S.H. (2018). Design of Fuzzy Logic Based Controller for Gyroscopic Inverted Pendulum System. Int. J. Fuzzy Log. Intell. Syst..

[21] Kajita S., Kanehiro F., Kaneko K., Yokoi K., Hirukawa H. The 3d linear inverted pendulum mode: A simple modeling for a biped walking pattern generation. Proceedings of the IEEE/RSJ International Conference on Intelligent Robots and Systems.

[22] Kajita S., Kanehiro F., Kaneko K., Fujiwara K., Harada K., Yokoi K., Hirukawa H. Biped walking pattern generation by using preview control of the zero moment point. Proceedings of the IEEE International Conference on Robotics and Automation.

[23] Collins S., Ruina A., Tedrake R., Wisse M. (2005). Efficient bipedal robots based on passive dynamic walkers. Science.

[24] Sugihara T., Nakamura Y., Inoue H. Realtime humanoid motion generation through zmp manipulation based on inverted pendulum control. Proceedings of the IEEE International Conference on Robotics and Automation.

[25] Li J., Chen W. (2011). Energy-efficient gait generation for biped robot based on the passive inverted pendulum model. Robotica.

[26] Kajita S., Tani K. (1995). Dynamic Biped Walking Control on Rugged Terrain Using the Linear Inverted Pendulum Mode. Trans. Soc. Instr. Contr. Eng..

[27] Kajita S., Hirukawa H., Harada H., Yokoi K. (2014). Introduction to Humanoid Robotics.

[28] Motoi N., Suzuki T., Ohnishi K. (2009). A Bipedal Locomotion Planning Based on Virtual Linear Inverted Pendulum Mode. IEEE Trans. Ind. Electron..

[29] Shibuya M., Suzuki T., Ohnishi K. Trajectory Planning of Biped Robot Using Linear Pendulum Mode for Double Support Phase. Proceedings of the 32nd Annual Conference of IEEE Industrial Electronics.

[30] Shibuya M., Sato T., Ohnishi K. Trajectory generation of biped robots using Linear Pendulum Mode with virtual supporting point. Proceedings of the 10th IEEE International Workshop on Advanced Motion Control.

[31] Luo X., Li W., Zhu C. (2011). Planning and control of COP-switch based planar biped walking. J. Bionic Eng..

[32] Luo X., Xu W.L. (2012). Planning and Control for Passive Dynamics Based Walking of 3D Biped Robots. J. Bionic Eng..

[33] Luo X., Zhu L.Q., Xia L. (2015). Principle and method of speed control for dynamic walking biped robots. Robot. Auton. Syst..

[34] Bruneau O., Ouezdou F.B. (1999). Distributed ground/walking robot interaction. Robotica.

[35] Kajita S., Morisawa M., Harada K., Kaneko K., Kanehiro F., Fujiwara K., Hirukawa H. Biped Walking Pattern Generator Allowing Auxiliary ZMP Control. Proceedings of the International Conference on Intelligent Robots and Systems (IEEE/RSJ).

[36] Park J.H., Kim E.S. (2009). Foot and Body Control of Biped Robots to Walk on Irregularly Protruded Uneven Surfaces. IEEE Trans. Syst. Man Cybern. B.

[37] Hong Y.D., Lee K.B. (2016). Dynamic Simulation of Modifiable Bipedal Walking on Uneven Terrain with Unknown Height. J. Electr. Eng. Technol..

